# 

**Published:** 1893-06

**Authors:** 


### CONTENTS.

ORIGINAL COMMUNICATIONS—
- Puerperal Septicæmia. By J. I. Darby, M. D., Americus, Ga. .... 193
- Chronic Gonorrhœa in the Male and Its Treatment. By Floyd W. McRae, M. D., Atlanta, Ga. .... 200
- Some Uses of Water as a Therapeutic Agent. By C. C. Stockard, M. D., Atlanta, Ga. .... 206
- Abortion in an Hour. By Chas. H. Harris, M. D., Cedartown, Ga. .... 211

SOCIETY REPORTS—
- Atlanta Society of Medicine .... 214

CORRESPONDENCE—
- New York Letter .... 228

EDITORIAL—
- The Journal and the Colleges .... 233
- Professional Education in the United States .... 234
- Encouraged .... 235

SELECTIONS AND ABSTRACTS—

- General Medicine and Therapeutics .... 237

- Eye, Ear, Nose and Throat .... 239

- Skin and Genito-Urinary Diseases .... 242

- Obstetrics and Gynæcology .... 246

- Surgery .... 248

MEDICAL ITEMS .... 251
BOOK REVIEWS .... 253
INDEX TO ADVERTISEMENTS .... xii
ITEMS OF INTEREST .... x, xi, xii, xxxi
PREMIUM OFFERS .... xv

